# Fatal *Leptospira* spp./Zika Virus Coinfection—Puerto Rico, 2016

**DOI:** 10.4269/ajtmh.17-0250

**Published:** 2017-06-26

**Authors:** Paige Neaterour, Aidsa Rivera, Renee L. Galloway, Myriam Garcia Negrón, Brenda Rivera-Garcia, Tyler M. Sharp

**Affiliations:** 1 Division of Vector-Borne Diseases, Centers for Disease Control and Prevention, San Juan, Puerto Rico;; 2 Division of High Consequence Pathogens and Pathology, Centers for Disease Control and Prevention, Atlanta, Georgia;; 3 Puerto Rico Department of Health, San Juan, Puerto Rico

## Abstract

Coinfection with pathogens that cause acute febrile illness (AFI) can complicate patient diagnosis and management. This report describes a fatal case of *Leptospira* spp./Zika virus (ZIKV) coinfection in Puerto Rico. The patient presented with a 5-day history of AFI; reported behavioral risk factors for leptospirosis; was diagnosed with possible leptospirosis, dengue, chikungunya, or ZIKV disease; and received appropriate treatment for leptospirosis and dengue. Following a 3-day hospitalization, the patient died due to acute gastrointestinal hemorrhage, and kidney and liver failure. Serologic diagnostic testing for leptospirosis and ZIKV disease was negative; however, molecular diagnostic testing performed postmortem was positive for detection of *Leptospira* spp. and ZIKV nucleic acid. This case demonstrates the need for continued clinical awareness of leptospirosis in areas experiencing outbreaks of pathogens that cause AFI and the need for evaluation of coinfection with AFI-causing pathogens as a risk factor for increased severity of disease.

## INTRODUCTION

Co-circulation of pathogens that cause acute febrile illness (AFI) complicates clinical diagnosis of patients, which can result in delays in initiating lifesaving medical interventions.^1,2^ Leptospirosis is a tropical AFI that is the result of infection with *Leptospira* species bacteria, which are spread through contact with the urine of infected animals including rodents, dogs, cattle, pigs, and sheep.^3^ Risk factors for infection with *Leptospira* spp. bacteria include exposure to open sewers and contaminated water, and close contact with rats or other animals.^3^ Common manifestations of leptospirosis include fever, headache, myalgia, vomiting, and thrombocytopenia.^4^ Roughly 10% of patients with leptospirosis will progress to life-threatening manifestations including pulmonary and gastrointestinal hemorrhage, and renal and hepatic failure.^4^ The annual worldwide burden of leptospirosis is estimated to exceed 1 million cases and 58,000 deaths.^5^ Recommended management of patients with suspected leptospirosis focuses on early administration of antibiotics, which can be lifesaving.^1,2^

In addition to outbreaks of leptospirosis, the recent emergence of Zika virus (ZIKV) in the Americas has further complicated the diagnosis of AFI.^6^ ZIKV is a flavivirus primarily transmitted by *Aedes* species mosquitoes, most frequently *Ae. aegypti*.^6^ Although most ZIKV infections are asymptomatic, common symptoms of ZIKV disease include rash, myalgia, arthralgia, and fever.^6^ Rare but severe manifestations associated with ZIKV infection include Guillain–Barré syndrome, severe thrombocytopenia, and encephalitis.^6–8^ Because neither a vaccine nor a specific therapy has yet been developed, prevention of ZIKV disease relies on avoidance of mosquito bites and treatment focuses on symptom management.^6^

Leptospirosis and ZIKV disease are both now common throughout much of the tropics including the Caribbean island of Puerto Rico.^5,6,9,10^ This raises concern for decreased recognition of leptospirosis cases, as leptospirosis has been overlooked during outbreaks of dengue and chikungunya.^10–13^ Although reports of coinfections with *Leptospira* spp., dengue virus, chikungunya virus, and ZIKV demonstrate the possibility of coinfection,^11,13,14^ testing for multiple pathogens is not always performed and hence it is unclear how frequently such coinfections occur. Without routine identification of such coinfections, assessment of potential increases in disease severity resulting from coinfection cannot be evaluated.

The following report describes a fatal case of *Leptospira* spp./Zika coinfection in Puerto Rico.

## CASE REPORT

**Figure 1.**
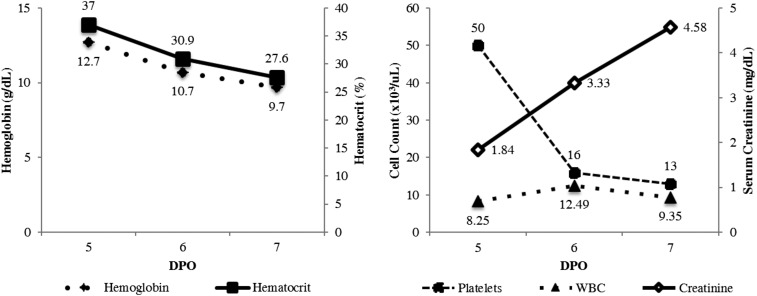
Laboratory data from a patient who died after coinfection with *Leptospira* species bacteria and Zika virus, Puerto Rico, 2016. DPO = days post-illness onset; WBC = white blood cell count. Reference ranges: hemoglobin = 12.5–16.6 g/dL; hematocrit = 37.9–51.1%; platelets = 160–374 × 10^3^ cells/µL; WBC = 4.31–10.68 × 10^3^ cells/µL; creatinine = 0.60–1.30 mg/dL.

In July 2016, a 48-year-old obese (body mass index = 31.2) male with no significant past medical history presented to the emergency department with a 5-day history of subjective fever, nausea and vomiting, diarrhea, headache, and myalgia. The patient denied the use of home medications, and reported growing banana plants at home, household exposure to rats, and exposure to a stray dog. On evaluation, the patient was afebrile, tachycardic (heart rate = 112 beats per minute), and normotensive (blood pressure [BP] = 111/74 mmHg). Physical examination was notable for scleral icterus and jaundice. Initial laboratory results revealed thrombocytopenia and elevated serum creatinine (Figure 1). The patient was given intravenous (IV) fluids, ranitidine, and promethazine, oral acetaminophen, and admitted to the hospital with a differential diagnosis of dengue, Zika, leptospirosis, and chikungunya.

On hospital day 2, the patient became febrile (temperature [T] = 38.7°C) and hypotensive (BP = 93/60 mmHg). Laboratory results demonstrated worsening thrombocytopenia, new onset leukocytosis, rising creatinine levels, and elevated liver enzymes (Table 1). Urinalysis was significant for large amounts of bilirubin and hematuria. An electrocardiogram (EKG) demonstrated an incomplete right bundle branch block, nonspecific T wave abnormalities, and possible left ventricular hypertrophy; however, a subsequent EKG revealed a normal ejection fraction, normal wall motion, and mild tricuspid and mitral regurgitation. A chest X-ray and abdominal ultrasound were unremarkable. Blood and urine cultures were drawn, and diagnostic testing was ordered for Zika, dengue, chikungunya, and leptospirosis. The patient was treated with IV ceftriaxone and oral acetaminophen.

**Table 1:** Laboratory results of a patient who died following coinfection with *Leptospira* species bacteria and Zika virus, Puerto Rico, 2016

	DPO		
Laboratory value	6	7	Reference range
AST (U/L)	484	604	15–37
ALT (U/L)	102	107	12–78
Total bilirubin (mg/dL)	10.1	14.59	0.20–1.00
Direct bilirubin (mg/dL)	NP	11.49	0.00–0.20
PTT (seconds)	NP	41.2	24.0–33.6

ALT = alanine aminotransferase; AST = aspartate aminotransferase; DPO = days post-illness onset; NP = not performed; PTT = partial thromboplastin time.

On hospital day 3, the patient was transferred to the intensive care unit (ICU) for severe thrombocytopenia, persistent hypotension (BP = 60/40), and onset of hematochezia suggestive of an acute gastrointestinal bleed. Cardiology and hematology/oncology teams were consulted and a dengue patient management protocol was initiated, including hourly monitoring of vital signs. The patient remained febrile (T = 39.6°C), and hepatitis, human immunodeficiency virus, and urine toxicology tests were ordered. Laboratory results showed worsening thrombocytopenia, decreasing hemoglobin, increasing creatinine, and increasing levels of liver enzymes. The patient’s hemodynamic instability and hemorrhagic manifestations were treated with a dopamine infusion, two units of packed red blood cells, and two units of platelets. He was treated in the ICU for 12 hours before suffering cardiac arrest and death. Cause of death was listed as hepatorenal syndrome. Autopsy was not requested.

In the serum specimen collected on hospital day 2/illness day 6, serology to detect anti-ZIKV and anti-*Leptospira* spp. IgM antibodies were both negative. Testing of the same serum specimen by reverse transcription-polymerase chain reaction was positive for detection of ZIKV nucleic acid with a cycle threshold (CT) value of 26 (positivity cutoff = 38), and negative for detection of dengue and chikungunya virus nucleic acid.^15^ Because of the clinical picture, additional diagnostic testing of the same specimen was performed by polymerase chain reaction (PCR) to detect *Leptospira* spp. bacteria, which was positive with a CT value of 34 (positivity cutoff = 40). Insufficient nucleic acid was available for multilocus sequence typing, and microscopic agglutination testing (MAT)^16^ was negative. All additional diagnostic testing and cultures were negative.

## DISCUSSION

This case demonstrates the importance for providers in Puerto Rico and throughout the tropics to be aware of the possibility of coinfection with the multiple pathogens that cause AFI, including *Leptospira* spp. and ZIKV. The patient described herein experienced a disease course consistent with severe leptospirosis including gastrointestinal hemorrhage and hepatic and renal failure.^4^ Although the patient did not demonstrate rash, which is a common sign of ZIKV disease,^6^ the proportion of patients with ZIKV infection who experience fever, myalgia, and headache in the absence of rash has varied between reports.^12^ Nonetheless, we cannot rule out that the patient was asymptomatically infected with ZIKV and that all observed signs and symptoms were attributable to infection with *Leptospira* spp. bacteria. Similarly, although the CT values corresponding to detection of *Leptospira* spp. and ZIKV nucleic acid observed in the patient’s serum specimen were within expected ranges, we cannot rule out the possibility of ZIKV infection altering the patient’s immune response to result in increased bacteremia or vice versa. Overall, it is difficult to confidently assess the relative contribution of ZIKV infection to the patient’s clinical course or fatal outcome.

This case also demonstrates the utility of molecular diagnostic testing to identify etiologic agents of AFI, as serologic testing was negative for both ZIKV and *Leptospira* spp. bacteria. Current methods for serologic testing include IgM antibody detection and MAT, the first of which has low sensitivity in the first week of illness and the second of which requires technical expertise and oftentimes a convalescent serum specimen for a definitive diagnosis.^17^ Conversely, molecular testing methods using PCR have been demonstrated to be more effective in the acute phase.^18^ Similarly, antigen-based detection methods such as the Dual Path Platform assay have been shown to be highly sensitive in the acute setting of severe disease and show promise for rapid detection in a broad range of healthcare settings.^19^ Future clinical-based studies should evaluate the prospect of *Leptospira* spp. molecular testing as a first-line diagnostic tool to improve patient care and outcome, as delayed initiation of antibiotic treatment and hospital admission have been associated with increased mortality.^1,2,10^

With the addition of ZIKV to the list of differential diagnoses for causes of AFI in patients in or recently returned from Puerto Rico and elsewhere in the tropics, there is a growing need to better understand both if and how coinfection may negatively impact health outcomes. Although there has only been one other documented case of *Leptospirosis* spp.*/*ZIKV coinfection,^14^ increased disease severity has been demonstrated in coinfections of flaviviruses and other pathogens (e.g., influenza and dengue viruses^20^). Further investigation should seek to quantify the incidence of similar coinfection cases, as well as evaluate if coinfection is associated with worse clinical outcome.
